# Troubleshooting the implementation of a template to evaluate and record SDF caries arrest

**DOI:** 10.3389/fdmed.2025.1694909

**Published:** 2025-11-14

**Authors:** Y. O. Crystal, S. G. Song, V. Saraza Reduta, M. Majstorovic, V. Raveis

**Affiliations:** 1 Department of Pediatric Dentistry, New York University College of Dentistry, New York, NY, United States; 2 Department of Orthodontics and Pediatric Dentistry, University of Maryland School of Dentistry, Baltimore, MD, United States; 3 Department of Cariology and Comprehensive Care, New York University College of Dentistry, New York, NY, United States

**Keywords:** SDF, caries arrest, record-keeping, caries management, electronic template, silver diamine fluoride

## Abstract

**Purpose:**

The aim of this study was to investigate the feasibility of using an electronic note template to evaluate and record caries arrest from the application of 38% Silver Diamine Fluoride (SDF) at the New York University College of Dentistry Pediatric Clinic (NYUCD-PD). The study evaluated adherence to its use and explored barriers and alternatives to document this procedure among the post-doctoral residents.

**Methods:**

A template was designed to collect baseline characteristics of treated teeth, application methods, and caries arrest at subsequent visits. The template was implemented among 26 post-graduate students on October 31, 2023. A retrospective chart review was conducted in March 31, 2025 to evaluate utilization of the template and extracted data was analyzed using descriptive statistics. The residents' feedback was collected during two focus-group sessions and analyzed qualitatively to assess their understanding of the importance of documentation, identify barriers to utilization of the template, and explore other alternatives for documentation.

**Results:**

392 visit encounters on 250 patients were analyzed. Template utilization increased with time from graduated PGY-2 residents to current residents, but it was not universal or uniform. Qualitative analysis revealed that residents fully understand the importance of accurate record keeping, and the template's intended advantages. However, barriers for utilization like time constraints, difficulties when reporting multiple lesions in our complex patient population, and its accessibility within the system, limit their capabilities to comply.

**Conclusion:**

Although a standardized electronic template can be an effective method of evaluating and documenting caries arrest in SDF-treated teeth, compliance with its use at every encounter is difficult in a hectic university clinic that treats very young children. Further investigation is required to overcome barriers for its use, and to test additional strategies that could be feasible in a dental educational setting.

## Introduction

Silver diamine fluoride (SDF) is a clear alkaline liquid medicament that is effective in achieving caries arrest in primary teeth (1–3). The most common 38% concentration Ag(NH_3_)_2_F available in the United States is used for arresting caries through remineralization and antibacterial action. Although the exact mechanisms of action are still being studied, we know that silver disrupts key bacterial functions and inhibits biofilm formation, while the fluoride remineralizes weakened dentin and enamel by forming fluoridated hydroxyapatite (3–5).

The application is generally well-tolerated, and is safe, effective and affordable, leading the American Academy of Pediatric Dentistry (AAPD) to support its use for caries arrest on primary teeth in children and adolescents as part of a comprehensive caries management plan (1, 6). Its teaching and utilization as an interim caries arrest treatment has become universal in Pediatric dental programs in the US (7).

Caries arrest after application of SDF is difficult to evaluate (8). A combination of several factors are used to determine whether or not a lesion has been arrested, including the size of the lesion, consistency on tactile examination, color change, and pre- and post-operative sensitivity (8, 9). A carious lesion can increase in size even though the color remains black, meaning that color alone is not a definitive determinant of arrest (8). Determining caries arrest is critical in evaluating the efficacy of SDF and requires consistent documentation of the factors and reassessment over time (9).

In most pediatric residency programs, patients are often treated by multiple resident providers who are at different stages of their training and are overseen by different faculty members who rely on the electronic notes of the electronic health records (EHRs) to follow and assess the continuity of patient care. The patient could be seen by several providers throughout the course of his treatment. Consistent documentation of the treatment provided (including application protocols and variables of caries arrest) is important to allow measuring the outcomes of treatment and ensuring high quality of care.

At the New York University Pediatric Dentistry Clinic (NYUCD-PD), there are currently several templates used to document different procedures, but not one to use as a standardized method of documenting lesion arrest after SDF treatment. We developed the electronic note template to be imported manually into AxiUm Scratchpad, outlining the affected teeth numbers and surfaces, and two SDF application protocols: 1 min application with micro brush or 10 s of application followed by 20 s of Light-Emitting Diode (LED) light curing. The first protocol follows current AAPD recommendations, including cleaning the carious tooth, adequate isolation, and scrubbing of SDF with a micro brush for 1 min (6, 10). The second protocol is being tested to evaluate if use of a dental curing light after SDF application improves arrest rates, as it is known that shorter application times and tooth location (posterior regions) result in lower rates of caries arrest (11, 12). An *ex-vivo* study utilizing the LED in dental light curing units (LCUs) at 520 mW/cm^2^ with a wavelength of 450–470 nm for 20 s after 10 s of SDF application was found to increase the precipitation of silver ions into infected dentin, compared to applying SDF for 1 min effectively resulting in a similar penetration into dentin as the 1 min control groups (13, 14). Light curing the SDF for 40 s after application also resulted in harder dentin compared to 1 min application without light curing (15). Standardized documentation of the baseline characteristics of the lesion, as well as the variables that can have an effect on arrest, can help determine the efficacy of each method in our clinic. The additional parameters collected to evaluate caries arrest include lesion size, consistency, color, and sensitivity. Our team designed a template with all the parameters to record baseline and post operative characteristics of a caries lesion that would allow for proper evaluation of arrest over time (8), as well as a record of treatment rendered (Supplementary Table S1).

Prior utilization of templates in EHRs has proven benefits in an educational setting. At the Kapi’olani Medical Center pediatric clinic in Honolulu, revising the intake documentation to assess dental history and fluoride vanish increased rates of fluoride varnish application (16). At the Pediatric Care Clinic at Children's Mercy Hospital in Kansas City, modification of record templates to include prompts for teeth and gums, more detailed dental history, and recorded fluoride varnish application improved documentation and classification of high-risk-for-caries patients, resulting in higher rates of fluoride varnish application and regular referrals to dental care providers (17). Dental schools can implement quality measurement processes in their clinical programs to improve the clinical practices and better prepare their graduates for future practice (18).

Our hypothesis is that residents will NOT be able to utilize the template to consistently record baseline lesion characteristics, application details and caries arrest variables in all encounters subsequent to SDF application due to system barriers.

The primary aim of this study was to evaluate compliance with the use of the template. The second aim was to determine potential barriers that could have prevented residents from utilizing the template as intended, explore options to overcome these barriers, and propose feasible alternatives to uniformly document and evaluate caries arrest resulting from SDF application.

## Materials and methods

This study's protocol was exempt from federal regulations as determined by the New York University Institutional Review Board under IRB study numbers i24-00328 for the retrospective chart review and FY2025-9845 for the focus groups sessions. A standardized AxiUm note template (Supplementary Table S1) was implemented after all 18 postgraduate pediatric dentistry residents at NYUCD received it via email on October 31, 2023. During a common session, all residents were introduced to the template, explaining its purpose and given specific instructions for how to complete it during clinical encounters that included SDF application. Its use was strongly recommended, but not mandatory. After the senior class graduated (9 residents) and a new group of 8 residents joined the program, they also received the template via email (August 2024) and were introduced and calibrated into its use. A total of 26 residents had access to the template during the study time.

A retrospective chart review of electronic health records (EHR) of patients who visited NYUCD-PC from October 1, 2023 to March 31, 2025, was conducted. Data was extracted from AxiUm for the patients who received treatment with 38% SDF, using the ADA CDT code D1354. The patient charts were de-identified and the following items were extracted from each record and every encounter: number of teeth treated, dates of treatment, tooth number, tooth surfaces, lesion size, color, caries consistency, tooth sensitivity, application method, extent of template utilization (full, partial or none), and reapplication at follow-up visits (yes or no). Clinical notes were reviewed to determine the final clinical status (outcome) relevant to previous history of the treated tooth and assess patient's behavior by means of Frankl scale.

Each resident was randomly assigned an individual number for de-identification during the evaluation. The number was matched with each encounter of SDF application and/or follow-up, as documented in the patients' charts. It was further documented if the resident made use of the template either completely, partially, or did not utilize it at each of the encounters. Percentages of total encounters of utilized templates were calculated, excluding and including partial utilization of the template.

To further explore the residents' experience with the utilization of the template, two focus group sessions were conducted on February 26, 2025; one for first-year residents and the other for the second-year residents. The purpose of these focus group sessions was to identify barriers to template usage and to explore alternative methods to overcome these barriers with the aim of improving evaluation and documentation of caries arrest. Written consent forms were obtained from all residents. The sessions were led by VR, with discussion facilitated by AD, VDSR, and SGS. Faculty members involved in the sessions (VR and AD) were not involved in the residents' program (to avoid residents' feeling of coercion) and their roles were intended to minimally interject into conversation, while ensuring a smooth discourse focused on specifically determined themes. Topics related to the themes concentrated on 12 seed questions (included in Supplementary Table S2) that pertained to each of three themes:

1. the importance of recording and evaluating caries arrest resulting from SDF application (in general, and via an AxiUm note template).
2. identifying barriers in preventing the template from being utilized, and
3. exploring alternative methods for assessing consistent evaluation/documentation of the treatment outcomes.

The sessions were audiotaped by utilizing Zoom H4n Pro 4-Track and Apple iPad Mini 5th generation portable recorders and transcribed verbatim for qualitative analysis. During the transcription process, residents' feedback was kept anonymous by replacing their names with the randomly assigned numbers. Only one of the study team members (VDSR) had access to the list of the assigned numbers and their corresponding resident. The rest of the evaluating team were blinded to who the comments came from, except for their year of residency (first year, or second year).

Constant comparative analysis (19) was used to qualitatively analyze themes in the data that emerged from the dialogue. In applying this analytic approach, each comment was reviewed and linked to a specific theme by authors VDSR and SGS working independently. Topics within the themes were identified, and comments were re-evaluated to fit into the themes. Topics and themes were continuously evaluated, and data was updated by adding residents' comments throughout the conversation during the entire session. The theme blocks were reviewed and streamlined into the final categories and revised until consensus was reached between the two original evaluators, and then with the whole team.

**Figure 1:**
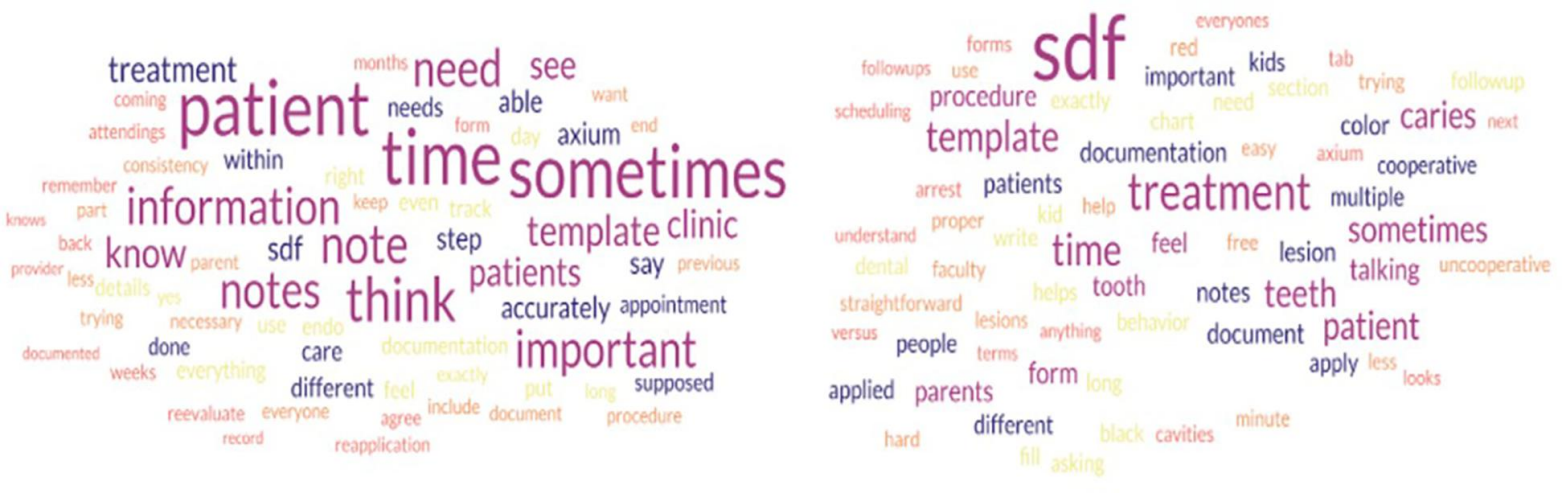
Word clouds representing frequency of words spoken during focus group discussions of PGY-1 (left) and PGY-2s (right).

**Figure 2:**
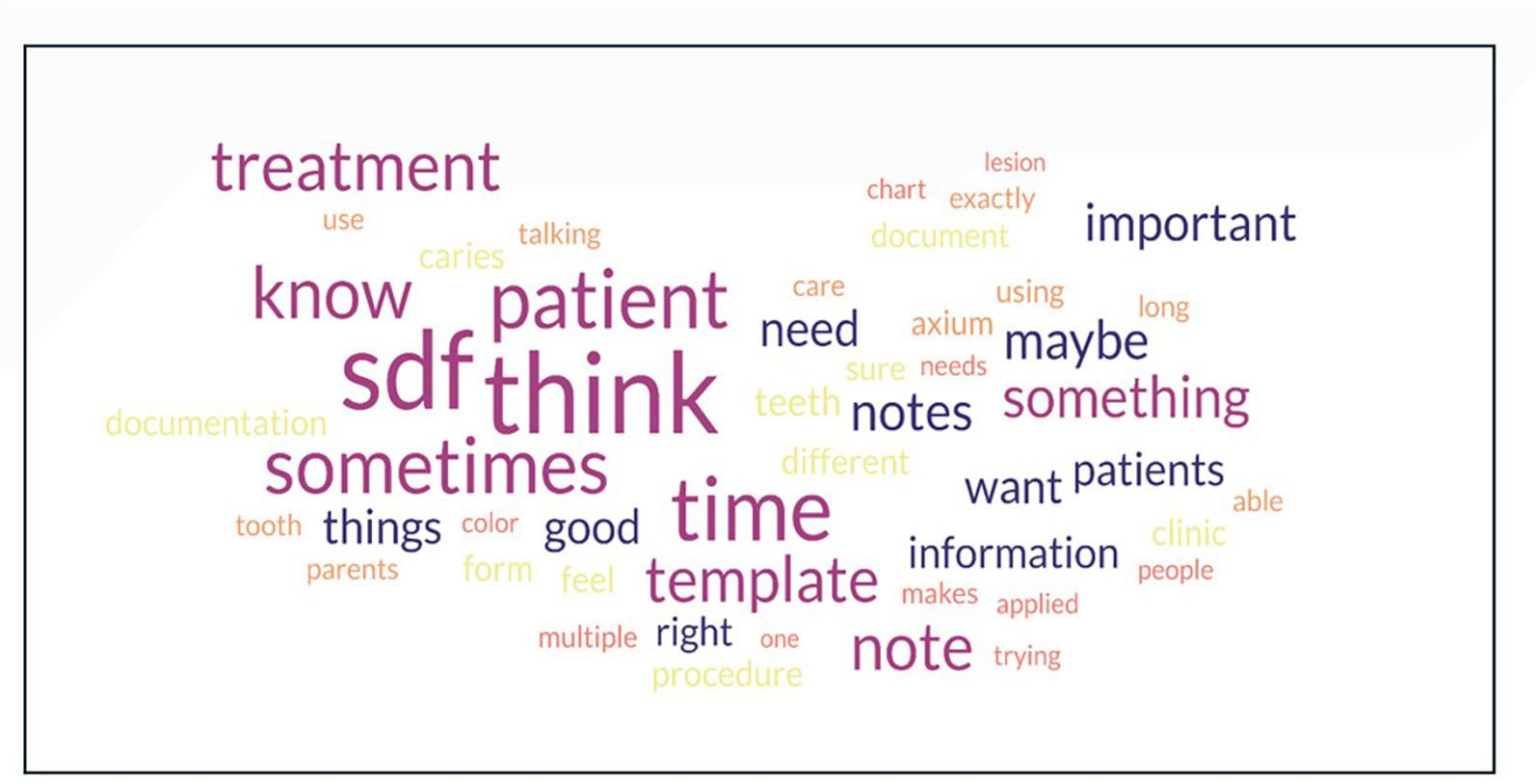
Word cloud with combining most frequent words on both focus groups.

In the final step, facilitator's comments were removed from the transcripts, and the scripts were placed into a Word cloud generator (https://www.freewordcloudgenerator.com/). Prepositions and filler words such as “in”, “um”, and “of” were removed as represented in Figures 1, 2.

## Results

Out of 294 total patient records, 32 were treated in a separate undergraduate clinic and therefore were excluded from the analysis. Twelve patients were treated in a satellite site outside of the NYUCD-PC where paper charting was utilized and were also excluded. One resident (special program and not part of the original 26) was also excluded as she was in a prior graduated class and was not present during calibration. A total of 373 visit encounters for 250 patients were analyzed. The follow-up period for treated patients varied from 0 (never returned for evaluation to the clinic) to 3 years, since some of the patients had received initial SDF applications prior to the dates of the study.

### Utilization of the template

Template utilization among 26 residents ranged from 0% (never used) to 100% (always used), with 13 residents utilizing the template in over 50% of all encounters and 13 residents using it less than 50% of all encounters (2 residents never used the template) (Supplementary Table S3). The number of encounters for the graduated class and current PGY-1 class was lower because they had been treating patients for only 8 months and 5 months respectively, while the current PGY-2 class were treating patients for the full 13-month period.

In many encounters, the template was not used in its entirety, but only partially, recording accurately only the treatment provided but not all variables, or variables for some teeth, but not all teeth treated. In the graduated class, when including counts for partial utilization of the template, only one person utilized the template in over 50% of all encounters. In the current PGY-2 class, 6 out of 9 residents used it in over 50% of all encounters and in the current PGY-1 class, 6 out of 8 residents used it in over 50% of all encounters. When excluding partial utilization of the template in the graduated class, no one utilized the template in over 50% of all encounters. In the current PGY-2 class, 5 out of 9 residents used it in over 50% of all encounters and in the current PGY-1 class, 5 out of 8 residents used the template in over 50% of all encounters (Supplementary Table S3).

### Focus groups and qualitative analysis

Seventeen pediatric residents (all residents) from the first and the second-year cohorts voluntarily participated in one of two focus group sessions. Residents provided comments about the SDF treatment they performed, and their experience using the template, thus enabling further insight into their perspectives. From the constant comparative analysis of their comments, several major themes emerged, such as the importance of record-keeping, barriers to template usage, alternative ways to document caries arrest, and miscellaneous comments that did not fit into any of the categories.

#### Theme 1. Importance of record-keeping

Qualitative analysis of the focus groups data revealed that both current PGY-1 and PGY-2 residents have a clear understanding of the importance of properly documenting the clinical status and outcomes. Patient-centered care was the factor that garnered the most comments. Residents understood the importance of tracking the efficacy of the treatment provided, noting changes in the patient's behavior, facilitating continuity of care, and the medical-legal consequences that may result from inconsistence or inappropriate documentation. It is important to note that NYUCD-PC patients are shared by all residents (due to multiple rotations in their schedule), so patients are assigned to any available resident on the day of their scheduled visit. Continuity of care provided exclusively by one resident is difficult to obtain. As one resident summarized, while agreeing with the group, “*It's a matter of standardization and consistency in terms of continuity of care. So, when we pass down our patients to each other, we know exactly where to look and how to interpret the information. So, it takes out a step from our mental processing of what happened at previous visits. So, mostly standardization and quality control to make sure things were properly done and are consistently documented.”* Residents also mentioned that documentation became especially important (and challenging) when a patient required multidisciplinary care across several departments and facilities, where different EHR systems (ex. AxiUm vs. Epic) can make accessing patient records even more difficult as more steps are required to gain access to a patient's records, using up precious clinic time. They also recognized that the challenge is compounded with the difficulties in scheduling a follow-up visit in a full-schedule clinic, when there is a high rate of no-shows (many broken appointments). This makes documentation all the more important for high quality patient-centered care, since sometimes visits are re-scheduled by front desk personnel, only based on the note in the EHR: “*I know I have to push whenever I try to get a 2-week follow-up and…be like please try to find a half an hour slot to squeeze it in somewhere. Sometimes I have to look at my patients specifically and be like maybe this procedure won't take that long and I can squeeze them in.”*

Residents also briefly stated that utilizing a template saves time, however, “*if [one is] going to go do something that's outside the guidelines or standard of care, [one has] to include his rationale for it. It's important to put that [rationale] into the documentation*.” Meaning that there may still be more information to add in addition to what is contained in the template to fully record important facts about the encounter that will help the following provider to offer optimal care.

#### Theme 2. Barriers to utilization of the template

Several barriers for template utilization emerged, specifically related to time constraints which were accentuated by patient behavior (cooperation for treatment), punctuality, and by treatment plan alterations that result in less time to perform the planned procedures and recordkeeping. Since the priority is to complete the treatment in the scheduled appointment time, recordkeeping gets relegated to just a few minutes between appointments, or to the end of the day when crucial details can be overlooked. Other barriers mentioned were technological (hardware) or equipment malfunctions that often result in decreased efficiency, and difficulties accessing the template within the software system. As one resident shared: “*During the appointment, we're trying to focus on the care of our patient, but we have delays in treatment, whether that'd be a patient showing up late or the keyboards malfunctioning. Also, a lot of our patients [and their parents] don't speak English where we have to use the interpreter…so our appointments are mostly time spent with the patient. Then when we find ourselves doing our notes, it's usually on our own laptops where it's a lot easier to type the note out, instead of having to go back on a computer in the clinic because it takes a long time to pull up the chart for the faculty to swipe it.*” Additionally, another resident shared: “*AxiUm is so essay-based where you're constantly typing everything out. But when we're rotating through the hospital and using Epic, there's short-cuts to particular templates that I can make my own notes on. It helps me change things really fast and it saves so much time.*” The difference between the time required to accurately report the encounter varies significantly between patients, based on the amount of treatment provided, language barriers, comorbidities, behavioral challenges during the visit and other factors. More specifically, for patients with severe early childhood caries: “*I think it just gets tricky though when you have multiple lesions that you need to apply SDF on. So it just takes a lot more time in order to implement everything*.” In general, residents are conflicted as they express their desire to document detailed notes. However, as the previous comments indicate, extraneous circumstances and factors sometimes prevent them from doing so.

#### Theme 3. Alternatives for documenting caries arrest

Many residents suggested various alternatives that could be used to improve the current system of template utilization. One of them was to use a separate form/charting method with its own tab built into AxiUm, that would be more accessible for faster and equally accurate documentation.

It was interesting that the use of clinical photographs was not favored as an option to monitor and identify caries arrest, as most of the residents agreed upon the importance of tactile examination. In addition, taking the clinical photographs (especially on posterior teeth) would add an additional task that most of the young children (who are the ones that require SDF), may have difficulty cooperating with. In addition, it was perceived that setting up for a clinical photo would add more technological complications. As one resident pointed out: “*With the intraoral cameras, we would need upgraded technology. We would actually need it integrated into our chairs, not something that we would have to check out and plug it in*.”

However, residents suggested that charting and color-coding SDF-applied surfaces on the odontogram prior to treatment would provide better visual reference. As one resident mentioned to much agreement from their peers: “*When you treatment plan SDF, there's no indication of the odontogram, you have to go to completed treatments and see where was the SDF and when was it placed. If maybe when you treatment plan it, it's a color on the tooth. Because having us chart caries that turns red on the chart as a finding because that's what I’ve had to do to indicate SDF is in that area.*” Another resident suggested a separate tab to consolidate all the SDF treatment notes to be more readily accessible so that “*each time you were doing the forms…you knew exactly how many times it was applied rather than having to read the note and see that reapplication was at this time*.”

However, it is crucial to note that even with all these suggestions, one of the most prominent challenges in a clinical setting is the difficulty of applying SDF in children exhibiting uncooperative or disruptive behavior during dental appointments. The American Academy of Pediatric Dentistry's SDF Chairside Guide suggests that a successful application of SDF requires adequate moisture control and a minimum contact time of one minute per lesion to achieve optimal therapeutic efficacy (20). However, in clinical practice—especially amongst children with high levels of dental anxiety, limited coping mechanisms, or developmental and behavioral conditions- they often demonstrate resistance to even the most basic intraoral procedures. Thus, providers may find themselves in situations where they are simultaneously attempting to behaviorally stabilize a child while trying to isolate the affected tooth, dry the surface, and apply the SDF with precision and for the recommended time. Even in cases where application is initiated, movement or saliva contamination may prematurely disrupt the one-minute application window, reducing the potential clinical effectiveness of the SDF. Such encounters are not only physically demanding but also clinically complex, especially with the allocated appointment window provided.

A summary of the quotes collected in the focus groups organized via continuous comparative analysis is presented on Supplementary Table S4.

### Tag cloud analysis

Figure 1 depicts two word clouds that were directly created from the transcriptions. In the combined word cloud (Figure 2), the most frequently used words were “SDF”, “think”, “time”, “sometimes”, and “patient”. For the current PGY-1 class, the more frequently used words were “patient”, “time”, “sometimes”, and “think”. For the current PGY-2 class, it was “SDF”, “treatment”, “template”, “time”, “sometimes”, and “caries”. “Time” and “sometimes” appeared in all three-word clouds, emphasizing that the barriers of time and non-uniformity of the encounters between patients are high in the residents' minds when describing delivery of care to their patients.

## Discussion

Results show that adherence to utilization of the template as a method for documentation was not ideal, confirming our hypothesis. 1 out of 9 of the graduated residents utilized the template for more than 50% of the encounters, compared to 7 out of 9 of the current PGY-2 class, and 7 of 8 for the current PGY-1 class. This clear trend towards increased usage may reflect improved awareness or engagement due to ongoing calibration efforts, greater familiarity with the AxiUm system, or a change in the overall culture of the clinic.

Electronic templates help to shorten the amount of time for documentation while maintaining an accurate description of the procedure completed. They also can serve as a blueprint for teaching to help residents review procedures ahead of time. However, the reality of implementation in a busy academic setting is complex and presents many barriers that have been reflected in the qualitative analysis.

Partial use of the template was present in 6.2% of encounters, as residents occasionally utilized the clinical protocol section of the template instead of the entire note. Findings from the chart review are consistent with what was reported during qualitative analysis of the focus groups data.

Results from the qualitative analysis confirm that the residents understand the rationale of documenting the completed procedure; citing its importance in tracking treatment outcomes, baseline characteristics and procedural details that can contribute to failures or successes; ensuring continuity between providers; and protecting the provider against legal risks. However, residents also verbalized importance in prioritizing quality patient care and obtaining informed consent within a limited time, resulting in less time to complete the documentation of the clinical notes of treatment. Lack of adherence to the template is multifactorial and influenced by environmental factors (i.e., technical difficulties with hardware and software), time constraints, and interpersonal factors (i.e., patient cooperation, delayed appointments, non-English-speaking patients, changes in treatment plan). These issues were compounded by charting outside of clinical hours (often on secure personal devices) thus making access to templates more cumbersome.

On the contrary, experiences with other electronic medical record (EMR) systems, such as Epic, proved to be more user-friendly due to customizable shortcuts and templates. This highlights an opportunity for system-level improvements in AxiUm to promote greater efficiency and ease of use. This migration is already planned for our university clinic.

It is worth noting that capturing a photograph of lesions each visit was not a popular solution. This could be because many children who receive SDF because they are very young and/or uncooperative, often resulting in difficulties sitting in the chair or tolerating a mirror and explorer. Placing an intraoral camera is not always plausible and could exacerbate time constraints and uncooperative behavior in addition to the additional technological (hardware and software) complications.

Instead, residents favored color-coding tooth surfaces on the odontogram to reflect SDF-treated teeth. This would provide an easy shortcut without having to read through notes dating back to possibly several years and provide a visual for teaching purposes during case discussions. However, arrested lesions sometimes become active. Perhaps a system of documenting arrested vs. non-arrested lesions and SDF-treated vs. non-SDF-treated lesions could be combined. For example, non-arrested carious lesions are currently highlighted in red in AxiUm. Perhaps a lighter shade of red to indicate that this lesion was active but was treated with SDF this visit. This can be repeated with a different color for un-arrested lesions. However, the downside of this method is that it relies on the assessment of an individual provider, and does not record the characteristics of a lesion that would allow to make accurate comparisons over time to confirm arrest.

 The most common words from the tag clouds (Figures 1, 2) were “SDF”, “think”, “time”, “sometimes”, and “patient” This perhaps highlights the cognitive dissonance residents feel while treating patients as they try to balance their efforts and time between high-quality patient care and providing accurate and detailed documentation while being constrained by time. This dissonance can contribute to feeling more stressed or “burned out” in the clinic.

### Limitations and strengths

Several limitations must be considered. The study was limited to one institution, one EHR program (AxiUm), and a relatively small cohort of residents, which limits generalizability. In contrast, all residents chose to participate in the study, so no selection bias was present. In addition, focus group sessions were run with no direct faculty members present and the recordings were stated to be confidential and anonymous, which encouraged residents to speak their minds. The limited time of the focus groups could have prevented more information from being collected. In addition, although focus-group lead (VR) verbally documented what was happening in the room (like some instances of consensus head-nodding), the recordings may have missed capturing other non-verbal messages that were being communicated.

In spite of the limitations, this study records and highlights common problems in the dental education system. Most pediatric post-doctoral programs in the US and abroad, service the same kind of population that we do, who face many barriers to accessing dental care for their children including insurance and economic constraints, language and transportation limitations, non-flexible work schedules, and more. The resulting high rates of broken appointments, late show-ups, and cases lost to follow-up, are universal in post-doctoral university clinics. As most programs share similar difficulties of technological challenges and time constraints, the information collected from the residents in this study will echo the comments that are shared in other residency programs. Finding solutions to these challenges to improve data collection and reporting is very important to provide optimal, effective patient-centered dental care.

In terms of transformative strategies and future directions, innovative solutions in reshaping clinical documentation and templates include the use of artificial intelligence, which already has been used and implemented in medical healthcare systems for the purpose of streamlining the documentation process and enhancing its accuracy and quality. Recording and transcribing using AI to populate key areas of procedure templates, not only can improve and customize the existing template draft but also add more personalized details of treatment and interactions with the patient, which are of utmost importance in the patient-centered healthcare approach (21). Ultimately, it can result in improvements in the recording and evaluation of treatment outcomes, while significantly reducing administrative time which providers may re-direct towards better quality healthcare. As with any specific and new methodology, details to be considered include: ethical and technical challenges, appropriate training and costs, feasibility of integration with the existing system, potential data safety issues, as well as bias when it comes to health disparities. But this can all be resolved with an appropriate and well-planned design, in which artificial intelligence supplements, not replaces human capabilities.

## Conclusions

An electronic note template is useful for standardized recording of caries arrest after SDF application when the patient is being treated by many different providers. However, universal utilization is problematic in a hectic academic clinic environment. Further investigation is required to test improvements that will allow to implement this method successfully and uniformly. Alternative methods of evaluating caries arrest and documentation including markings on the existing odontogram in AxiUm, or a pre-existing EHR form could achieve accurate evaluation and documentation of caries arrest across the lifespan of the treated tooth and should be tested for feasibility and efficacy.

To achieve better quality of care and allow for accuracy in follow ups and assessment of all treatment outcomes, standardization and uniformity in collecting documentation are essential in educational settings. Ultimately, developing and adopting transformative strategies will be essential to advance clinical documentation practices and enhance patient care. Therefore, it is of utmost importance to troubleshoot the current methods and explore other avenues of assessing and documenting not only caries arrest through SDF application, but all treatment provided to improve the health of the population we serve.

## Data Availability

The raw data supporting the conclusions of this article will be made available by the authors, without undue reservation.

## References

[1] Crystal YO Niederman R. Evidence-based dentistry update on silver diamine fluoride. Dent Clin N Am. (2019) 63(1):45–68. 10.1016/j.cden.2018.08.011 30447792 PMC6500430

[2] Care EO. Safety Data Sheet–Advantage Arrest Silver Diamine Fluoride 38% (2015). Available online at: https://www.elevateoralcare.com/site/images/AASDS082415.pdf (Accessed November 05, 2025).

[3] Mei ML Lo ECM Chu CH. Arresting dentine caries with silver diamine fluoride: what’s behind it? J Dent Res. (2018) 97(7):751–8. 10.1177/0022034518774783 29768975

[4] Lansdown AB. Silver. I: its antibacterial properties and mechanism of action. J Wound Care. (2002) 11(4):125–30. 10.12968/jowc.2002.11.4.26389 11998592

[5] Chu CH Lo EC. Microhardness of dentine in primary teeth after topical fluoride applications. J Dent. (2008) 36(6):387–91. 10.1016/j.jdent.2008.02.013 18378377

[6] Crystal YO Marghalani AA Ureles SD Wright JT Sulyanto R Divaris K Use of silver diamine fluoride for dental caries management in children and adolescents, including those with special health care needs. Pediatr Dent. (2017) 39(5):135–45. PMID: 29070149 29070149

[7] Crystal YO Janal MN Yim S Nelson T. Teaching and utilization of silver diamine fluoride and hall-style crowns in US pediatric dentistry residency programs. J Am Dent Assoc. (2020) 151(10):755–63. 10.1016/j.adaj.2020.06.022 32979954 PMC7510543

[8] Fontana M Khera D Levy S Eckert G Katz B Yanca E A randomized clinical trial to assess caries arrest by using silver diamine fluoride in U.S. children: interim findings. Pediatr Dent. (2024) 46(1):8–12. Available online at: https://pmc.ncbi.nlm.nih.gov/articles/PMC10921985/ PMID: 38449039 38449039 PMC10921985

[9] Neuhaus KW Kühnisch J Banerjee A Martignon S Ricketts D Schwendicke F Organization for caries research-European federation of conservative dentistry consensus report on clinical recommendations for caries diagnosis paper II: caries lesion activity and progression assessment. Caries Res. (2024) 58(5):511–20. 10.1159/000538619 38684147 PMC11446318

[10] Horst JA Ellenikiotis H Milgrom PL. UCSF protocol for caries arrest using silver diamine fluoride: rationale, indications and consent. J Calif Dent Assoc. (2016) 44(1):16–28. 26897901 PMC4778976

[11] Yan IG Zheng FM Gao SS Duangthip D Lo ECM Chu CH. Effect of application time of 38% silver diamine fluoride solution on arresting early childhood caries in preschool children: a randomised double-blinded controlled trial protocol. Trials. (2022) 23(1):215. 10.1186/s13063-022-06130-1 35292085 PMC8922752

[12] Fung MHT Duangthip D Wong MCM Lo ECM Chu CH. Randomized clinical trial of 12% and 38% silver diamine fluoride treatment. J Dent Res. (2018) 97(2):171–8. 10.1177/0022034517728496 28846469 PMC6429575

[13] Karnowakul J Punyanirun K Jirakran K Thanyasrisung P Techatharatip O Pornprasertsuk-Damrongsri S Enhanced effectiveness of silver diamine fluoride application with light curing on natural dentin carious lesions: an *in vitro* study. Odontology. (2023) 111(2):439–50. 10.1007/s10266-022-00755-z 36269519

[14] Crystal YO Rabieh S Janal MN Cerezal G Hu B Bromage TG. Effects of LED curing light on silver diamine fluoride penetration into dentin. J Clin Pediatr Dent. (2023) 47(6):44–50. 10.22514/jocpd.2023.071 37997234

[15] Toopchi S Bakhurji E Loo CY Hassan M. Effect of light curing on silver diamine fluoride in primary incisors: a microscopic ex vivo study. Pediatr Dent. (2021) 43(1):44–9. PMID: 33662250 33662250

[16] Johnson SC French GM. A quality improvement project to optimize fluoride varnish use in a pediatric outpatient clinic with multiple resident providers. Hawaii J Health Soc Welf. (2020) 79(5 Suppl 1):7–12. Available online at: https://pmc.ncbi.nlm.nih.gov/articles/PMC7260867/32490379 PMC7260867

[17] Okah A Williams K Talib N Mann K. Promoting oral health in childhood: a quality improvement project. Pediatrics. (2018) 141(6):e20172396. 10.1542/peds.2017-2396 29802117

[18] Hunt RJ Ojha D. Oral health care quality measurement and its role in dental education. J Dent Educ. (2017) 81(12):1395–404. 10.21815/jde.017.099 29196327

[19] Weinstein ND. The precaution adoption process. Health Psychol. (1988) 7(4):355–86. 10.1037//0278-6133.7.4.355 3049068

[20] Chairside guide: silver diamine fluoride in the management of dental caries lesions. Pediatr Dent. (2018) 40(6):492–517. Available online at: chrome-extension://efaidnbmnnnibpcajpcglclefindmkaj/https://www.aapd.org/media/Policies_Guidelines/R_ChairsideGuide.pdf PMID: 32074924 32074924

[21] Zaretsky J Kim JM Baskharoun S Zhao Y Austrian J Aphinyanaphongs Y Generative artificial intelligence to transform inpatient discharge summaries to patient-friendly language and format. JAMA Netw Open. (2024) 7(3):e240357. 10.1001/jamanetworkopen.2024.0357 38466307 PMC10928500

