# Supplementary material for: Troubleshooting the implementation of a template to evaluate and record SDF caries arrest
Source: Front Dent Med. 2025 Nov 14;6:1694909. doi: 10.3389/fdmed.2025.1694909 (PMC12660238; doi:10.3389/fdmed.2025.1694909)
Supplement: Supplementary file 1 [file Table1.docx]

**Table 1: AxiUm template for documentation sent to all post-graduate residents via email and asked to utilize it for each visit and follow-up.**

**SDF INITIAL VISIT:**

SDF treatment protocol: Applied Vaseline onto patient’s skin, lips and gingiva. Used [bite block/molt prop/foam bite block] and cotton roll isolation. Washed and dried cavitated surfaces using air-water syringe and gauze. SDF applied with microbrush on cavitated surfaces [tooth # and surface] for [10 seconds and LED cured for 20 seconds each] or [1 minute]. Discussed post-op instructions with parent.

Tooth# ____ Surface(s): O/I/B/F/M/D/L (refer to odontogram)

-Size (list the teeth): S: M: L:

-Color: Bl: Br: Y:

-Consistency: H: S: Mix:

Pre-op sensitivity: Y/N

**SDF FOLLOW UP:**

1) For lesions not arrested

SDF treatment protocol: Applied Vaseline onto patient’s skin, lips and gingiva. Used [bite block/molt prop/foam bite block] and cotton roll isolation. Washed and dried cavitated surfaces using air-water syringe and gauze. SDF applied with microbrush on cavitated surfaces [tooth # and surface] for [10 seconds and LED cured for 20 seconds each] or [1 minute]. Discussed post-op instructions with parent.

Tooth# ____ Surface(s) O/I/B/F/M/D/L (refer to odontogram)

-Size: (list teeth) S: M: L:

-Color: Bl: Br: Y:

-Consistency: H: S: Mix:

Reapplication at this visit:  Y/N

Post-op sensitivity: Y/N/increased/decreased

2) For lesions arrested

Tooth# ____ Surface(s) arrested O/I/B/F/M/D/L (refer to odontogram)

-Size: (list teeth) S: M: L:

-Color: Bl: Br: Y:

-Consistency: H: S: Mix:

Reapplication at this visit:  Y/N

Post-op sensitivity: Y/N/increased/decreased
