# Supplementary material for: Troubleshooting the implementation of a template to evaluate and record SDF caries arrest
Source: Front Dent Med. 2025 Nov 14;6:1694909. doi: 10.3389/fdmed.2025.1694909 (PMC12660238; doi:10.3389/fdmed.2025.1694909)
Supplement: Supplementary file 2 [file Table2.docx]

**Table 2. Focus Group Guide. Includes the seed questions centered around three central themes used to guide the focus group sessions.**

**FOCUS GROUP GUIDE**

**Focus Group Themes to explore**

1. Do the residents understand the importance of recording treatment to be able to evaluate the outcome of it? Do they understand what it means for the patient? Do they see the reason behind trying to use templates for consistent recording/revaluation? Do they feel that patient centered care is adequately emphasized in their education? Do they feel that this activity is important to improve the educational goals of the department and the experience of future residents? Does their prior educational experience affect their views?
2. What were the barriers for implementation? Do they have suggestions for improvement?
3. Can they think of alternative ways to achieve the same purpose? Consistently record to evaluate the treatment regardless of who is the provider?

**Focus Group Tentative Questions to address the Themes**

**Questions for theme A**

1. Why do you think a template to evaluate caries arrest was designed?
2. Do you feel that you have an idea of the success of the treatment you provide? Is it important to you?
3. Do you think that patient centered care can be a top priority in an educational environment like this?
4. What was your experience on this topic in your prior education (dental school)?

**Questions for theme B**

1. How was the experience of utilizing the SDF template?
2. What are some strengths of the template?
3. What are the barriers that prevented you from using the template?
4. How can the template be improved?
5. Any recommendations for improvement for template utilization?
6. What could the program do to overcome these barriers?

**Questions for theme C**

1. Can you think of other ways to be able to assess caries arrest or progression in a reliable way that could be implemented in our clinic?
2. Do you think photographs would work to do this? Can you foresee any hurdles with this method?
