# Supplementary material for: Troubleshooting the implementation of a template to evaluate and record SDF caries arrest
Source: Front Dent Med. 2025 Nov 14;6:1694909. doi: 10.3389/fdmed.2025.1694909 (PMC12660238; doi:10.3389/fdmed.2025.1694909)
Supplement: Supplementary file 3 [file Table3.docx]

| **Resident** | **# of Non Usage Encounters** | **# of Partial Usage Encounters** | **# of Complete Usage Encounters** | **% Encounters Where Complete Template was Used** | **% Encounters Where Complete OR Partial Template was Used** |
| --- | --- | --- | --- | --- | --- |
| **1** | 14 | 0 | 5 | 26.32 | 26.32 |
| **2** | 9 | 0 | 6 | 40 | 40 |
| **3** | 6 | 0 | 5 | 45.45 | 45.45 |
| **4** | 7 | 0 | 3 | 30 | 30 |
| **5** | 15 | 0 | 0 | 0 | 0 |
| **6** | 7 | 1 | 1 | 11.11 | 22.22 |
| **7** | 8 | 0 | 5 | 38.46 | 38.46 |
| **8** | 13 | 0 | 5 | 27.78 | 27.78 |
| **9** | 6 | 1 | 5 | 41.67 | 50 |
| **10** | 0 | 1 | 25 | 96.15 | 100 |
| **11** | 15 | 2 | 9 | 34.62 | 42.31 |
| **12** | 5 | 2 | 41 | 85.42 | 89.58 |
| **13** | 9 | 1 | 16 | 61.54 | 65.33 |
| **14** | 13 | 0 | 18 | 58.08 | 58.06 |
| **15** | 2 | 7 | 7 | 43.75 | 87.5 |
| **16** | 7 | 0 | 3 | 30 | 30 |
| **17** | 10 | 1 | 7 | 38.89 | 44.44 |
| **18** | 7 | 4 | 19 | 63.33 | 76.67 |
| **19** | 3 | 1 | 3 | 42.86 | 57.14 |
| **20** | 3 | 0 | 1 | 25 | 25 |
| **21** | 1 | 0 | 2 | 66.67 | 66.67 |
| **22** | 3 | 0 | 5 | 62.5 | 62.5 |
| **23** | 4 | 0 | 0 | 0 | 0 |
| **24** | 0 | 0 | 3 | 100 | 100 |
| **25** | 2 | 0 | 2 | 50 | 50 |
| **26** | 0 | 1 | 2 | 66.67 | 100 |

**Table 1. Residents and frequency of template usage. Each row represents a resident. Different colors represent PGY-1s (green), PGY-2s (pink), and graduated PGY-2s (yellow). Darkened rows represent that the resident utilized the template in over 50% of encounters.**
