# Supplementary material for: Troubleshooting the implementation of a template to evaluate and record SDF caries arrest
Source: Front Dent Med. 2025 Nov 14;6:1694909. doi: 10.3389/fdmed.2025.1694909 (PMC12660238; doi:10.3389/fdmed.2025.1694909)
Supplement: Supplementary file 4 [file Table4.docx]

**Summary of Focus Groups’ quotes organized via continuous comparative analysis**

Table 4. Residents’ comments from Focus Groups’ transcriptions organized by Theme and Topic. PGY-1 (colored green) and PGY-2 (colored red).

| **Theme A: Importance of Record-Keeping** | **Recording Importance** | Just to know exactly how you did something, so you can see if there needs to be any improvements in the future. Like if you don’t have a record of it, how are you supposed to gauge how well you did something or did something correctly if you missed a step. I think it’s important to have a record. |
| --- | --- | --- |
|  |  | Just to piggyback off that, to see if things succeeded and if there were failures in the long run. |
|  |  | Also to add to that, I think unfortunately in our clinic we don’t have a lot of continuity of care so it’s really important that what we’re doing is very well documented so that the next provider knows exactly what we did and what needs to be done after. |
|  |  | I would branch off further and say that when you’re licensed as well, beyond the clinic, for lawsuits that’s protecting you. Consistency too and seeing your notes. |
|  |  | For every encounter to be honest. |
|  |  | When you’re talking to a parent, if the parent is unhappy of a service, it’s important to document the interaction between yourself and the parent. Also from a medical legal standpoint, if parents come and say something, just for your records as well to see what the encounter was. |
|  |  | Follow-up and seeing the arrested caries - that’s number one. Also, I’ve had kids come in who are too young to sit for treatment so we do SDF and it helps a lot. |
|  |  | And it’s very important to document how the behavior was, so at the next visit, you’re prepared. |
|  |  | So I think documentation is important for 1) calibration. Also, since we’re not always seeing the same patients every time and there’s different providers that are treating the same patient, in this case with SDF and the follow-ups, I think having proper or detailed documentation helps standardize the result that we get or the treatment we give. |
|  | **Reason Behind Template** | Probably because note taking wasn’t very good and to have a standardized, organized procedure. |
|  |  | Also to evaluate if multiple applications were needed and potential risk factors associated with that. |
|  |  | I agree with the group. It’s a matter of standardization and consistency in terms of continuity of care. So when we pass down our patients to each other, we know exactly where to look and how to interpret the information. So it takes out a step from our mental processing of what happened at previous visits. So mostly standardization and quality control to make sure things were properly done and are consistently documented. |
|  |  | So that everything’s more structured, organized- |
|  |  | Calibrated |
|  |  | Maybe help the next clinician or the next person, reading the note to know “oh we applied SDF there” so that’s all. |
|  |  | I know that there’s more materials that we’re able to use now and it’s not maybe studied as much so we want to see how it’s going especially with kids who are cooperative versus not cooperative, kind of putting more force on minimally invasive dental techniques so there’s that. |
|  |  | Maybe because there are children that we are treating are coming in with more caries |
|  |  | I think that people can word things differently and so when you are trying to understand exactly what something looks like from reading it, not from seeing it, it helps when there’s a template because everyone’s using the same procedure. |
|  |  | Everyone’s describing what they see in the same way so it’s easier to differentiate |
|  | **Patient-Centered Care** | Or it’s not filled out. Sometimes I feel like people can also assume the template is all that you should be including in your note and a lot of times there’s a lot more that you should include in your note rather than just going based off the template. That involves what material you used, step by step procedure, some sort of interaction or communication with the patient. |
|  |  | Going off that, I feel like even though there are templates, some of them are not utilized accurately. Not to call out the endo department, but sometimes their information is not easily accessible or legible. So it just goes to show that even if you do have a template, it may not be accurately used. |
|  |  | If you’re going to go do something that’s outside the guidelines or standard of care, you have to include your rationale for it. It’s important to put that into the documentation. |
|  |  | Also for our patient’s too, if they need access to this information or are interested in what we’re doing, the records also them to gain insight on what we did during that appointment. |
|  |  | Have clear consistent notes. Not just your physician notes, but also contact notes. Insurances on file and have a good excel sheet of appliances on file so you can check those records in order to see the history of the patient. Not just treatment notes. |
|  |  | Everytime we’re reviewing a patient, sometimes I also forget to do that. But looking at all the previous notes, I’ll try to decipher them. But if it’s written really clearly, it really helps. But I think that sometimes we don’t have time. |
|  |  | I guess from my experience, I know we have a PG and UG clinic. Sometimes, for instance, I had a patient that was first seen there [UG] and then they wanted to refer to endo but then endo the appointment in endo never got made. Then the tooth was never taken care of. Then finally they were brought to us [PG] as a walk-in patient. I was able to see it because I was on time, but then it got punted to him and then I don’t know what happened after. It’s so many points of contact with different departments and with us. Sometimes it’s like everyone has their own direction that they’re going at and then it gets spread out so far that it inhibits any type of movement forward. |
|  |  | That’s why it’s important to maintain documentation so that regardless of where the patient is going, you’re able to accurately keep track of the patient and what they need to be seen for, whether it’s at this clinic or that clinic. Because right now, here we have Axium and at Bellevue we have Epic. Epic is a lot more inclusive and utilized at different hospitals, we can still see the patient’s chart and see that they were seen for separate care at this hospital, on this day, this is the provider. Then we can reach out to that provider. So it’s a lot easier at Bellevue to use Epic as you’re able to track the patient. Here with Axium, for example, ortho or endo uses their own template within Axium. It’s not highly visible to everyone else, you have to go to a different part of it. But it’s important that the documentation is all there so that your patient accurately track them. |
|  |  | I feel like there’s different levels of of training within each department too and exposure to different education. So it goes off of why a template is important and communication between different departments between seniors, juniors and dental students is great in my opinion. |
|  |  | As we know that SDF causes the black staining and as it relates to the patient-centered care, knowing that there might be psychological impacts of having that so even though it helps caries arresting, it might not be as aesthetic so being mindful of telling parents that. You know, there is that chance that as we are monitoring it and as they get more cooperative and older, switching that out for something that’s white like a white filling, things like that kind of makes it more patient-centered, if that’s what the patient wants. |
|  |  | Well as a clinician offering patient-centered care, I think it’s important to have a variety of different approaches to treatment. So I think SDF is a viable component to that. It’s another tool that we have that we can utilize for our patients if it’s appropriate and as long as the patients understand the risks and benefits and the pros and cons to the treatment. But then, if we are able to inform them of all of that and use it as a resource to maybe buy us more time to do treatment or to stabilize a lesion without treatment, I think it’s beneficial to have as an option. |
|  | **Prior Educational Experience** | Also transfer of data too. Like when you’re transferring from your office to a physician's office. |

|  |  |  |
| --- | --- | --- |
| **Theme B: Hurdles** | **Barriers** | Some people think that just because you apply it once, no matter how many times you explain to them that this needs to be applied again, or that other things need to happen in order to keep the patient caries-free, they still don’t understand that and still don’t come back. |
|  |  | No barriers |
|  |  | I think it just gets tricky though when you have multiple lesions that you need to apply SDF on. So it just takes a lot more time in order to implement everything |
|  |  | The template itself is easy to fill out, it’s very straightforward. |
|  |  | I think because in the template, we’re either assigned for either one full minute or to scrub for 10 seconds and then light cure for 20, so I think we’re loosely assigned which one to do which sometimes, if a patient needs multiple teeth or even very uncooperative, I think it’s definitely harder to stick to the one minute if that’s what you’re assigned. |
|  |  | So in regards to the use of the template, I think it’s very straightforward. It’s not time-consuming at all. It asks all the questions that we would have to write in our note anyways so it makes it more streamlined for us, in my opinion. In regards to delivery of treatment, we did talk about some factors or barriers to render the treatment. I do think the light curing portion could be a little bit more technique sensitive. So I think that’s my opinion. Like scrubbing it versus light curing. |
|  |  | Can I add something? Sometimes it’s maybe not enough time but along with that sometimes it could be that we don’t remember everything. Sometimes we don’t have time to do our notes because clinic can get extremely hectic and we’re going from screaming child and there’s a lot going on in the session that sometimes when we do have the time to work on our note, we may not fully remember every single detail from the encounter because I’m not doing it right after. So it’s not that we’re not opting to include the information, it’s just that we genuinely just don’t remember. |
|  |  | I think part of that to is that when we are in those situations where seeing a screaming child, we’re also stressed out. That’s a more physiological response where you’re so worried about the moment, tunnel visioning. |
|  |  | Sometimes it’s like I’ll make my own assumptions too, oh if this kid has very clean teeth, no cavities, then the snacking would be guessed. |
|  |  | I think not only are you battling all that, sometimes when you’re running out of time, you have to think what are the most important things that I need to document right now versus things that I can document later that won’t affect the treatment. Meaning that I only have 10 minutes until the faculty has to go, I have to do this form and this form, but I’m going to miss part A and B and going to fill that out later or next time because there isn’t enough time. |
|  |  | I think there’s obviously within the appointment time where we’re trying to focus on the care of our patient, but we have delays in treatment, whether that’d be a patient showing up late, the keyboards are malfunctioning, a lot of our patients don’t speak English where we have to use the interpreter, we want to make sure our patients are accurately receiving the information that we’re telling them. So our appointments are mostly time spent with the patient and so when we find ourselves doing our notes, it’s after the fact and usually on our own laptops where it’s a lot easier to be able to type the note out, instead of having to go back and type the note on a computer in clinic and that takes another however long to pull up the chart in order for the faculty to swipe it. |
|  |  | I would say it’s a challenge. Similar to what someone said earlier about what they did if the keyboard is not working, then I have to come log into my computer hoping I don’t get logged out and then trying to make a note before Axium kicks me out again. That’s going to affect the details I put in my note. Or if it’s the end of the day and the attendings are trying to go home and they keep popping in and ask, “Are you done yet?” That’s going to affect the details I write in my note. We all can agree that’s a consistent thing that happens to us. “Are you done? Are you done?” then you want me to include all the details, and then they get mad at us if the details are not there. Something has to give. Maybe we need more time carved out in our appointment slots to ensure that these notes are accurate and in detail as they want. I don’t know if you guys agree with that. |
|  |  | Some attendings are a lot more proactive in wanting to read word on the note, making sure that everything is accurately documented, making sure you’re not missing anything versus there’s other attendings that trust you to think that you’re going to have everything written down. Usually the attendings that don’t read the note, usually those are the notes that are less in detail maybe. |
|  |  | That brings an interesting point because Axium is so essay-based where you’re constantly typing everything. But then at Bellevue, there’s smartphrases that I can make my own note and swap that in and helps me change things really fast. Notes are still comprehensive and includes everything important that I did but it saves so much time. |
|  |  | The template is always very important. Sometimes it seems that has way too much information that may not be necessary or relevant on exactly what we did that day, but that’s going to take away from being able to be extremely detailed in areas that we need to be just because at the end of the day, dentistry is a lot of about efficiency as well. We’re expected to do a lot in a short amount of time. Efficiency is key. I will say a lot of our treatment template seems that there’s a lot of information that may be important for the comprehensive exam or sixth month evaluation or entire treatment plan for the patient. But, I’m not sure all of the information is necessary for treatment specific tooth and filling it out is obviously going to take time from adding more details in an area that might require it. So maybe shifting the dynamic to allow for more pertinent information to be filled in. |
|  |  | Also, faculty will direct you on what to do and change treatment plans and that also changes your consistency of the notes, the direction of where the treatment is going, how much time you’re delaying treatment. That’s a big thing. |
|  |  | I definitely feel if we are not documenting correctly, it’s hard to keep track of any changes on patients. I know that a lot of times occlusion is skipped by a lot of people. For me, it’s important because when I’m trying to evaluate the patient thoroughly, whether it’d be ortho or whatever it might be. Little pieces of information that should be in there even though it’s part of the template, it’s not put in. Makes you lose track of the application. You don’t know whether the procedure before or after has changed. Ortho will do their own comprehensive, but for us, how do you note if there’s a change? How can we present our patient if we don’t have all of the information we should have? I know I can be lazy to take BMI. I’ll take the height and weight of the patient but to go and get the BMI, that’s just an extra step that sometimes you’re just really crunched on time. So you don’t necessarily have to eliminate the information but figure out which information is the most prevalent. |
|  |  | Materials. Our keyboards are not working, our computers are timing out. I will write a much shorter note and probably document a lot less especially when I can’t get a single keyboard to work. |
|  |  | I think also timing of the patients, especially when they’re tardy. It forces us to rush through things especially if our other patient is here. So it’s tough. I don’t want to say it or admit it, but sometimes, I just end up going to the most important things. So time is really important. I’m on time but then patients need to be on time too. |
|  |  | I definitely feel if we are not documenting correctly, it’s hard to keep track of any changes on patients. I know that a lot of times occlusion is skipped by a lot of people. For me, it’s important because when I’m trying to evaluate the patient thoroughly, whether it’d be ortho or whatever it might be. Little pieces of information that should be in there even though it’s part of the template, it’s not put in. Makes you lose track of the application. You don’t know whether the procedure before or after has changed. Ortho will do their own comprehensive, but for us, how do you note if there’s a change? How can we present our patient if we don’t have all of the information we should have? I know I can be lazy to take BMI. I’ll take the height and weight of the patient but to go and get the BMI, that’s just an extra step that sometimes you’re just really crunched on time. So you don’t necessarily have to eliminate the information but figure out which information is the most prevalent. |
|  |  | You put on the SDF for as long as you can and then just finish because you lose them. So sometimes it’s not the full minute or 20 seconds or whatever, sometimes it’s less than that because of patient cooperation, so we just change that on the note |
|  |  | And partly why we chose this treatment for most of the time is because behavior is so uncooperative and when that happens, that affects possibly, I know when I am documenting, that also affects how I write the note. Because if it takes longer to apply the SDF, I have a shorter amount of time to write the note and the computer system. I know I need to get swipes from faculty. So there’s a lot of things environmentally, where the treatment of the patient affects the utilization of the template of proper documentation, etc, etc. |
|  |  | I do think that if it’s the last patient of your day and you’re running late and you have a lot of other things to do, you’re more harried and trying to make things as succinct as possible and might be more inclined to delete some things off the template. |
|  |  | We have the template in our notes section of Axium and we just copy and paste and just fill in what we have to fill in afterwards. It’s super easy |
|  |  | It’s pretty straightforward. |

|  |  |  |
| --- | --- | --- |
| **Theme C: Improvements** | **Suggestions/Alternative Ways** | Needs to be fully integrated and fully functioning with added tech support, which we don’t have right now. |
|  |  | The technology is also not there. We may be given all these fancy tools but we don’t have the systems to support it. |
|  |  | Yes |
|  |  | That’s a really good idea. |
|  |  | And with that, maybe even an alert mentioning hey this patient needs a follow up on this. Right now we’re just very reliant on looking at previous notes, which is good and we do that, but things get lost a little bit when it’s a very long note. |
|  |  | Yeah |
|  |  | Along with that, I think the alert would be good because also sometimes just our scheduling we’re booked out so we might not be able to bring the patient back within the amount of time for SDF reapplication. As a result, the process of applying it can get lost so an alert would be good so everyone who’s on the chart knows that this patient had SDF and they still need a re-application. Where are we now between the last application? |
|  |  | It’s a standardized form that’s on a tab in Axium to fill out. |
|  |  | Would it be hard if there were multiple lesions involved? |
|  |  | There should be a way to input multiple of them. |
|  |  | I think that’s such a good idea because sometimes it just gets lost in the sauce. I will say, when it comes to SDF, they never have an appointment available for us to see them when we’re supposed to for a follow up. It doesn’t defeat the purpose obviously because when they come again, we can still re-apply but they’re not coming soon enough. They’re coming much later than when they’re supposed to be coming in for their second application. |
|  |  | We’re still waiting on our renovation |
|  |  | Honestly the template is pretty helpful and when I see previous notes, I was able to clearly tell the lesions that were working and whether it needs to be reapplied. I think a more centralized form, like an SDF tab. So everyone knows the dates when it was applied or have a drop down for color or consistency. It makes it quicker and efficient. |
|  |  | Template is also good because you’re doing something the same way each time because it has however many seconds or minutes and you’re not doing it based on what you think, but rather going step by step based on accurately needs to happen. |
|  |  | Yes |
|  |  | To be honest, no. Because the SDF turns black whether it arrests or not so we need to feel for the hardness of it. Can give you some insight for sure but I don’t think that’s the necessary insight knowledge that we need to get a clear answer if we need to reapply it or not. I think that comes from tactile. Sure it would be helpful, but I don’t think that’s necessary to have. |
|  |  | I guess the intraoral would be good for what he saw that day because you can record keep that, but then you need tactile and other information or if patient reported pain or something like that over time and the follow up so you can assess better. |
|  |  | But with the intraoral cameras, we would need upgraded technology. We would actually need it integrated into our chairs, not something that we would have to check out and plug it in. |
|  |  | Or use our cell phones. |
|  |  | But we can see lesion progression, like if it’s getting bigger you can see it on a clinical photo. Because now we don’t always do the follow-up or we don’t always remember. |
|  |  | But color with SDF is not indicative of caries arrest. |
|  |  | Or get a diamond tester to see the hardness of the surface. |
|  |  | Or get a caries indicator. |
|  |  | I think a tab would be nice then you could document each time you were doing the forms so you knew exactly how many times it was applied rather than having to read the note and see that reapplication was at this time. Because sometimes people don’t code for the second reapplication of the SDF. |
|  |  | When you treatment plan SDF, there’s no indication of the odontogram, you have to go to completed treatments and see where was the SDF and when was it placed. If maybe when you treatment plan it, it’s a color on the tooth. Because having us chart caries that turns red on the chart as a finding because that’s what I’ve had to do to indicate SDF is in that area. |
|  |  | Especially now that there’s a code for it too, so when you’re updating the odontogram anyway, you just do dental treatments and that could have an associated color. |
|  |  | And also the red is showing that it’s an active lesion, but when we do the SDF, then it's now not active. So switching from the red to the yellow to show that it was active and now it’s arrested. It’s easy. |
|  |  | I thought of another idea. So instead of making a new form for the SDF and all the teeth, color and size because it takes too long, maybe in the notes section, like how they do ortho separately, maybe you can add an SDF section so you can put all the SDF notes. Like anytime an SDF note is written, it goes into one section of Axium so you can click on it and see all the previous notes related to SDF. |

|  |  |  |
| --- | --- | --- |
| **Miscellaneous** | | It’s a scheduling thing, we just don’t have space in our clinic. |
|  |  | It makes it delayed. |
|  |  | We’re scheduled out for months in advance. |
|  |  | Yeah we’re supposed to re-evaluate within a couple of weeks but we end up having to re-evaluate in a couple of months sometimes. |
|  |  | Maybe, I don’t know if it’s something that we can implement into our clinic, but maybe set aside slots for patients that need re-application of SDF so we know it’s being applied at the correct timing. |
|  |  | Yeah I know I have to push whenever I try to get a 2-week follow-up and I have to push to be like please try to find a half an hour slot to squeeze it in somewhere. Sometimes I have to look at my patients specifically and be like maybe this procedure won’t take that long and I can squeeze them in. |
|  |  | And we normally have someone that’s on emergency and yes, sometimes they’re really busy with emergency walk-ins but sometimes they’re not. So maybe it would be possible to put in a 15 minute slot for re-application of SDF. |
|  |  | Yeah, but then it kind of bleeds into creating space and where we can put it. |
|  |  | Yeah, it’s a front office management issue. |
|  |  | Sure, yeah they’re going to forget about it. But not that they’re going to forget about it, but also if you’re supposed to re-evaluate something in say 2-weeks that sounds more pressing rather than come back in 2 months. You’re going to remember something in 2-weeks but if it’s in 2 months then it might be less likelihood of them coming in. It’s not necessarily a fact but I can see that as seeming to a patient that it’s less pressing than come back in 2 weeks so that we can re-evaluate it. |
|  |  | Yeah |
|  |  | Maybe even for the parent, it saves them more visits down the line if we arrest the cavity now rather than waiting longer down the line and now it’s fully cavitated and SDF isn’t going to work anymore. |
|  |  | Yeah I agree. |
|  |  | Yeah. |
|  |  | Personally, I didn’t participate much because I feel like these days I see a lot of patients whose moms start refusing the SDF from the beginning. So it’s like half and half. Some are like “oh I want to try it on” but some are like “I don’t want to do it cause it’s going to be black”, like my kids are getting bullied and even if we tell all the pros and all the benefits, they just start being like “I don’t want it because my other kid had it already and then it was bad.” |
|  |  | I think that, when talking about the long-term, I know that there’s a lot of kids that come in, that once they recognize they have cavities, I’ve heard oh they saw their older sibling have so many cavities and then when they have cavities, they stop eating sweets now and brush their teeth all the time now. Sometimes, not saying you need to give them the black on the front for them to notice it and want to make that change, but that could have a lifetime thing of “oh, they see that that was cavities, what I was doing before was bad and want to improve on it from an oral health standpoint or even just making sure they come for dental appointments right? I think that sometimes too, going back to that initial thing with the parents. If you mention to them that it could potentially, if you continue following your recall appointments, getting the kid comfortable, like it doesn’t have to be a permanent thing on their front teeth. Well first of all, they’re going to exfoliate but then also there’s an option to do more treatment once they’re more cooperative, right, to remove the black staining and to replace it with something else and that could be a nice motivator to get them to be more on top of oral care for kids and families. |
|  |  | I think going off of that, like when they see the sibling has had extractions or something, and parents are more likely to go through the SDF because they don’t want their kid to go through a traumatic or more extensive procedure. So I think that helps a lot as well. |
|  |  | Something I also want to mention that hasn’t been talked about is that some faculty may administer SDF differently than it is written in the template and because of that, there might be differences in the procedure that we do compared to what our attendings are telling us to do. |
|  |  | So some faculty will put SDF between the teeth with floss, which is not included in the template. Some faculty will apply it like it’s supposed to so yeah. |
